# Filtration-induced production of conductive/robust Cu films on cellulose paper by low-temperature sintering in air

**DOI:** 10.1098/rsos.172417

**Published:** 2018-07-04

**Authors:** Shintaro Sakurai, Yusuke Akiyama, Hideya Kawasaki

**Affiliations:** Faculty of Chemistry, Materials and Bioengineering, Kansai University, 3-3-35 Yamate-cho, Suita 564-8680, Japan

**Keywords:** copper ink, air-atmosphere sintering, paper electronics, flexible devices

## Abstract

Cellulose paper is an attractive substrate for paper electronics because of its advantages of flexibility, biodegradability, easy incorporation into composites, low cost and eco-friendliness. However, the micrometre-sized pores of cellulose paper make robust/conductive films difficult to deposit onto its surface from metal-nanoparticle-based inks. We developed a Cu-based composite ink to deposit conductive Cu films onto cellulose paper via low-temperature sintering in air. The Cu-based inks consisted of a metallo-organic decomposition ink and formic-acid-treated Cu flakes. The composite ink was heated in air at 100°C for only 15 s to give a conductive Cu film (7 × 10^−5^ Ω cm) on the cellulose paper. Filtration of the Cu-based composite ink accumulated Cu flakes on the paper, which enabled formation of a sintered Cu film with few defects. A strategy was developed to enhance the bending stability of the sintered Cu films on paper substrates using polyvinylpyrrolidone-modified Cu flakes and amine-modified paper. The resistance of the Cu films increased only 1.3-fold and 1.1-fold after 1000 bending cycles at bending radii of 5 mm and 15 mm, respectively. The results of this study provide an approach to increasing the bending stability of Cu films on cellulose paper.

## 1. Introduction

Printable and flexible electronics have garnered considerable attention over the past 10 years because of their broad application in devices with substantial societal impact, such as flat displays, optoelectronics, radio-frequency identification tags, sensors and flexible electronic devices for the ‘internet of things’ community [1–3]. A wide variety of conductive inks to meet the performance requirements of these devices have been developed. These inks produce conductive films on flexible substrates with low heat tolerance, including metal (Ag, Cu) nanoparticles, carbon, conductive polymers and metal ion (Ag, Cu) complexes [1–6]. Among these inks, metal-based inks have advantages because of their high conductivity. Ag-based inks are the most prevalent because of their low electrical resistance and good oxidation resistance [7–11]; however, they involve expensive precursors and suffer from migration effects.

Recently, Cu-based inks have been investigated because of their low cost and low migration tendency. In general, thermal sintering of Cu-based inks is conducted under a reducing gas (e.g. H_2_ or vaporized formic acid) or inert gas (e.g. N_2_) to produce the conductive patterns because the air-thermal sintering process of Cu-based inks tends to oxidize the copper [12–32]. The development of air-sintered Cu-based inks compatible with quick and effective roll-to-roll processes in printable electronics would represent a substantial advancement. To this end, intense pulsed light (IPL) processes have been developed to sinter Cu nanoparticles under air ambient conditions [10,33,34]. Such IPL processes are very attractive for air sintering of Cu-based inks; however, these approaches require expensive and specialized equipment, and a drying process is usually needed before the sintering procedure. Recently, we developed an air-sinterable Cu-based ink compatible with low-temperature sintering at 150°C via simple oven heating [35]. The Cu-based ink was composed of 1-amino-2-propanol (AmIP)-stabilized Cu nanoparticles (approx. 3 nm size) and sub-micrometre Cu particles (approx. 0.3 μm size) to produce conductive Cu films with a resistivity of 70 µΩ cm on flexible polymer substrates. However, the instability of sub-10 nm Cu nanoparticles against oxidation and aggregation, which are inherent features of Cu nanoparticles, remains an issue.

Cellulose paper has been attracting interest as a substrate for paper electronics such as sensors, energy storage devices, transistors, flash memory and photovoltaic cells because paper substrates have obvious advantages such as good flexibility, biodegradability and composability in addition to being inexpensive and eco-friendly through paper recycling. Paper also has light weight and foldable character for portable electronics. However, the literature contains few reports on robust/conductive Cu films deposited onto paper using Cu-based inks because the micrometre-sized porous character of cellulose paper, which is composed of a layered fibrous network, makes their deposition difficult. To reduce the micrometre-sized porous character of cellulose paper used for metal-nanoparticle-based inks, paper precoating methods have been investigated [36–40]. We recently reported a composite ink of Cu-based metallo-organic decomposition (MOD) ink (i.e. copper salt) and micrometre-sized Cu flakes to produce conductive Cu films on cellulose paper [41]. The use of the MOD ink has the advantage of avoiding the instability associated with Cu nanoparticles (i.e. oxidation and aggregation). However, the air-thermal sintering process of this Cu-based composite ink makes the production of conductive Cu films difficult because of oxidation of the Cu film and degradation of paper during the air-sintering process. Therefore, our next goal is to fabricate a Cu-based ink capable of forming a conductive Cu film on cellulose paper in air.

Here, we present air-sinterable Cu-based composite ink to produce conductive Cu films on cellulose paper at low sintering temperatures in air. This work is the first report of conductive Cu film formation on paper using a Cu-based ink and low-temperature sintering in air. The air-sinterable Cu-based inks consist of a MOD ink and formic-acid-treated Cu flakes. The MOD ink is composed of Cu(II) formate, AmIP, oxalic acid and polyol solvents. The advanced feature of this approach is that the low-temperature heating of the Cu-based composite ink at 100°C in air for only 15 s using a low-cost halogen lamp can result in a conductive Cu film on cellulose paper. The self-reducing action from formic-acid-treated Cu flakes and the MOD ink produced the conductive Cu film on cellulose paper in air. The filtration of the Cu-based composite ink by cellulose paper provides accumulated Cu flakes on the paper, resulting in a sintered Cu film with high bending stability and few defects. In addition, we further found an effective strategy to increase the bending stability of sintered Cu film on cellulose paper through surface modification of both the cellulose paper and the Cu flakes.

## 2. Material and methods

### 2.1. Chemicals and materials

Whatman™ grade-3 qualitative filter paper (particle retention greater than 6 µm) and Whatman™ grade-4 qualitative filter paper (particle retention greater than 20 µm) were used as cellulose paper substrates for sintering the Cu films. Unless otherwise specified, the type of the paper discussed in this work corresponds to the grade-4 filter paper. Polyimide (PI) film was acquired from DuPont-Toray Co., Ltd, Japan. Copper(II) formate tetrahydrate (98%), AmIP (98%), ethylene glycol (99.5%), glycerol (97%), polyvinylpyrrolidone K30 (PVP) and methanol (99.5%) were purchased from Wako Chemicals, Japan. 3-Aminopropyltriethoxysilane (APTS, 98%) was purchased from TCI, Japan. Micrometre-sized Cu flakes (1050YP) with sizes of 1–2 µm were purchased from Mitsui Mining & Smelting Co. Ltd, Japan. All chemicals were used as received without further purification.

### 2.2. Preparation of copper inks

The Cu(II)–AmIP complex was prepared by mixing copper formate (2.5 g) and AmIP (2.55 ml) in methanol (7 ml); the resultant mixture was then stirred magnetically for 2 h. Free formic acid and residual methanol were removed under vacuum for 1 h at 50°C and then under vacuum for 24 h at room temperature (approx. 23°C), after which a solid complex was obtained.

The Cu flakes were immersed in formic acid for 1 h to remove the surface Cu oxide layers from the Cu flakes and were then separated by centrifugal filtration at 6000 r.p.m. for 10 min to obtain formic acid-treated Cu flakes. For strengthening the bending stability of sintered Cu film on cellulose paper, we modified the above procedure as follows. The Cu flakes (1 g) were immersed in 10 ml of formic acid in the presence of PVP (0.1 g) for 1 h. Then, the resultant solution was separated by centrifugal filtration at 6000 r.p.m. for 10 min to obtain PVP-modified Cu flakes. An amine-modified cellulose paper was prepared as follows: APTS (100 µl) was dissolved in an 80/20 (v/v) mixture of ethanol/water (10 ml). In the aqueous environment, APTS can be hydrolysed to form silanol groups, which are able to react with hydroxyl groups onto paper surfaces [42]. A piece of cellulose paper was immersed in the resulting solution for 2 h, and the excess solvent of the amine-modified paper was evaporated at 40°C for 3 h under reduced pressure. The obtained amine-modified paper was further heated at 110°C for 3 h, followed by thorough washing with ethanol and drying at room temperature.

The Cu-based composite ink was prepared by grinding the solid complex with a given amount of Cu flakes, the Cu(II)–AmIP complex, oxalic acid and ink solvent (mixture of 79 µl ethylene glycol, 79 µl glycerol and 6 mg oxalic acid) in a mortar in air until the mixture became an ink. The Cu-based composite inks were prepared using a weight ratio of Cu flakes to Cu(II)–AmIP complex of 3 : 1 (0.9 g: 0.3 g) according to a previously optimized protocol to produce low-resistance Cu films [38]. Oxalic acid was added to the composite inks to suppress Cu oxidation during the thermal sintering process in air. The optimized amount of oxalic acid in the composite ink was 0.5 wt%, which resulted in the minimum resistance value (electronic supplementary material, figure S1). The Cu-based composite ink was preserved at room temperature in air, and the resistivity of the sintered Cu film prepared from the Cu ink was almost unchanged even after two weeks (electronic supplementary material, figure S2). The viscosity of the Cu-based composite ink was measured using an RST Plus controlled stress rheometer (RST-CPS, Brookfield, USA). The viscosities of the ink were 600 mPa s and 150 mPa s at shear rates of 10 s^−1^ and 1000 s^−1^, respectively. The viscosities of the present inks make them applicable as screen-printing paints under operating conditions [43].

### 2.3. Characterization

The composite ink was deposited onto the PI or cellulose paper substrates using a doctor blade. The samples were then heated at different temperatures in air using a halogen lamp (Moisture Analyzers MF-50, A&D Co., Japan). Note that a gas was generated by the thermal decomposition of Cu(II) formate. Therefore, a glass-fibre sheet was placed underneath the paper substrate to facilitate the gas release during the heating process. The sheet resistance values of the Cu film after sintering were measured using a four-point probe instrument (Loresta AX MCP-T370, Mitsubishi Chemical Analytech Co., Japan). The electrical resistivity of the Cu film was evaluated from its thickness and its sheet resistance value. The thickness of the Cu film after heating was measured using a digital micrometer (MDH-25M, Mitutoyo Co., Japan), which indicated a thickness of 16 ± 2 µm. The thickness of the Cu film was similar to the thickness values obtained from scanning electron microscopy (SEM) observations of cross sections (18 ± 3 μm). We therefore used the film thickness measured using the digital micrometer in subsequent calculations. SEM images of the sintered Cu film were collected using a field-emission scanning electron microscope (JSM-6700, JEOL, Japan) operated at an acceleration voltage of 5.0 kV. An X-ray diffractometer (D2 Phaser, Bruker, Germany) equipped with a Cu-K*α* radiation source (*λ* = 1.5406 Å) was used to obtain X-ray diffraction (XRD) patterns of the sintered Cu film. Thermal analysis was conducted using a thermogravimetric–differential thermal analysis (TG–DTA) instrument (Thermo Plus EVO, Rigaku, Japan) at a heating rate of 10°C min^−1^ under flowing air. Analyses by Auger electron spectroscopy (AES) were carried out using a VG ESCA 220i-XL imaging X-ray photoelectron spectrometer. These measurements were conducted using monochromated Al-Kα X-rays (hν = 1486.6 eV) with a photoelectron collection angle of 90° with respect to the surface plane.

### 2.4. Bending tests

Bending tests on the sintered Cu films were conducted under repeated tensile and compressive loading on a custom-made bending tester [44]. The bending tester consisted of a clasp to hold the Cu films in position on the substrates and a moving part to bend the Cu films using an electronic motor. The bending tests were conducted at a frequency of 1 Hz, and a four-point probe was used to measure the changes in sheet resistance after 1000 cycles.

## 3. Results and discussion

### 3.1. Thermal behaviour and resistivity of air-atmosphere-sintered Cu films prepared from Cu-based composite inks

At low temperature (100°C) and under an air atmosphere, we deposited air-sinterable Cu-particle-based composite ink (mixture of sub-10 nm Cu nanoparticles and sub-micrometre Cu particles) previously reported to produce sintered Cu films on cellulose paper [35]. However, the Cu film on the paper showed high resistance (approx. 10^−2^ Ω cm) when heated at 100°C for 15 s in air. More recently, we reported a composite Cu-based ink composed of the MOD ink and micrometre-sized Cu flakes to produce conductive Cu films on cellulose paper under a nitrogen atmosphere [41]. This Cu-based composite ink resulted in sintered Cu films with improved conductivity because the combination of the MOD ink with Cu flakes caused filtration-induced accumulation of Cu flakes on the paper surface. Nevertheless, this ink still resulted in Cu films with high resistance (approx. 10^−3^ Ω cm) on paper when sintered at 100°C for 15 s in air because the Cu film oxidized during the air-sintering process. Thus, these previously reported inks could not produce sintered Cu films with low resistance (approx. 10^−5^ Ω cm) on paper via an air-atmosphere sintering process. For the Cu-based ink composite of the MOD ink and micrometre-sized Cu flakes, the Cu flakes were pretreated with formic acid to remove surface oxidation and inhibit the oxidation of the Cu film during the air-sintering process in this study. The formic acid protection of the Cu flakes was confirmed by the Fourier transform infrared spectrum (electronic supplementary material, figure S3).

**Figure 1:**
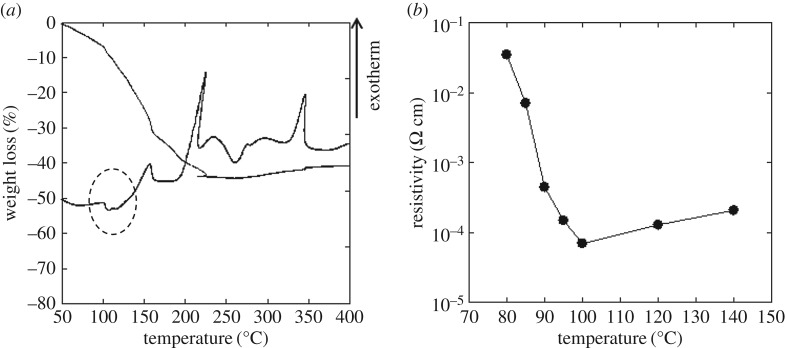
(*a*) TG–DTA curves of the composite ink under an air atmosphere and heated at 10°C min^−1^. (*b*) Electrical resistivity of Cu films on cellulose paper, as prepared from the composite ink and sintered at various temperatures for 15 s in air.

The thermal behaviour of the present Cu-based composite ink was examined by TG–DTA under air, as shown in figure 1*a*. The TG curve shows a gradual weight loss between 80°C and 200°C. The endothermic peak and the subsequent exothermic peak at approximately 100°C (the area indicated by the dotted oval in the figure) are assigned to the decomposition of the Cu(II)–AmIP complex and the subsequent reduction to metallic Cu, respectively. The TG–DTA data suggest the possibility of an air-atmosphere sintering temperature of 100°C for the composite ink. The exothermic peak above approximately 200°C is probably associated with the oxidation of copper in air because a weight increase is observed in this temperature range. Figure 1*b* shows the electrical resistivity of the Cu films on cellulose paper prepared from the composite Cu ink at different temperatures (80–140°C) in air for 15 s. The resistivity of the Cu film (greater than 10^−2^ Ω cm) was high at a sintering temperature of 80°C; however, the minimum resistivity of the film was 7 × 10^−5^ Ω cm at 100°C, where decomposition of the MOD ink occurs, as shown in the DTA curve in figure 1*a*. The resistivity values increased with increasing sintering temperature above 120°C because of the enhanced oxidation of copper in air at higher temperatures.

**Figure 2:**
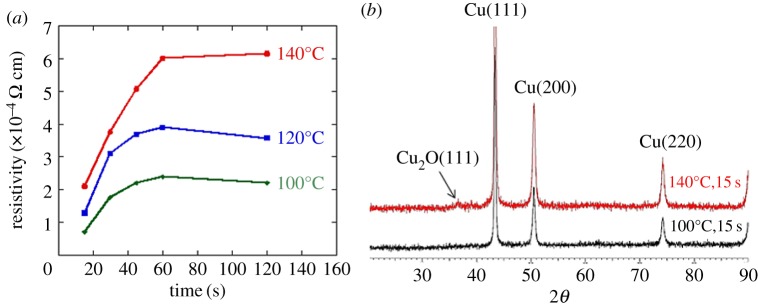
(*a*) Electrical resistivity of Cu films on cellulose paper as a function of heating time at 100°C, 120°C and 140°C for 15 s in air. (*b*) XRD patterns of sintered Cu films at 100°C and 140°C from the composite ink for 15 s in air.

Optimizing both the sintering temperature and the heating time is important to produce highly conductive Cu films under an air atmosphere. The sintering time of 15 s resulted in the lowest resistivity of the Cu film on cellulose paper, whereas the resistivity was too high to be measured for the specimen sintered for 10 s. Prolonged heating for more than 15 s increased the resistivity values at sintering temperatures of 100°C, 120°C and 140°C because of oxidation of the surface in air, as shown in figure 2*a*. The XRD pattern of the Cu film sintered at 100°C for 15 s in air shows only metallic copper peaks, whereas the pattern of the sample sintered at 140°C for 15 s shows a peak attributable to oxidized Cu (figure 2*b*); this oxidation led to the high resistivity of the Cu film. On the basis of the aforementioned results, we found that the present Cu-based composite ink can produce a conductive Cu film with resistivity of 7 × 10^−5^ Ω cm on cellulose paper at a low sintering temperature of 100°C for 15 s in air.

**Figure 3:**
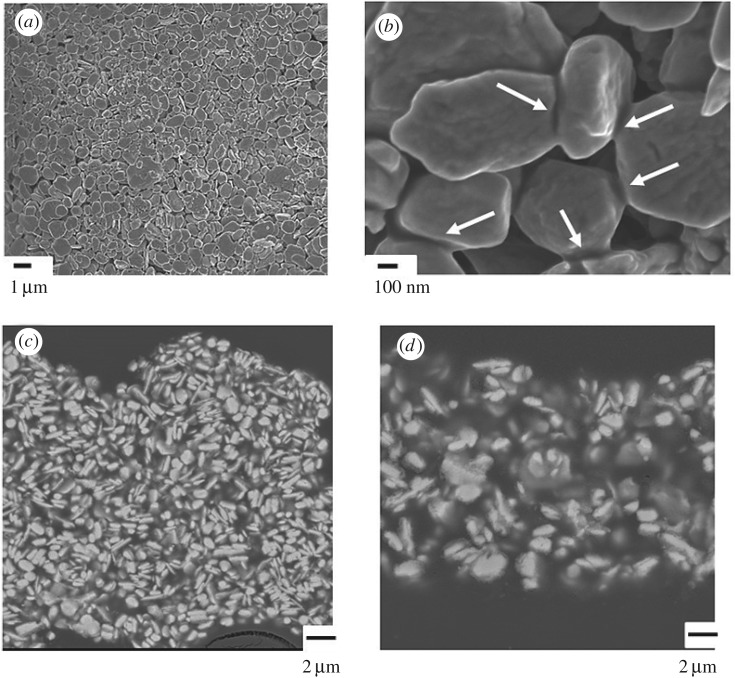
(*a*,*b*) SEM images of a Cu film on cellulose paper, as formed from composite Cu ink and heated under an air atmosphere at 100°C for 15 s. (*c*,*d*) SEM reflection images of a cross section of the sintered Cu films prepared at 100°C for 15 s in an air atmosphere on paper and PI substrates, respectively.

Figure 3*a* shows the SEM images of the Cu film on cellulose paper, as generated from the composite ink and sintered at 100°C for 15 s in air. These images show that the Cu flakes stacked together on cellulose paper. The expanded SEM image demonstrates the connection of Cu flakes (indicated by white arrows in figure 3*b*), which is caused by the copper from the thermal decomposition of the Cu(II)–AmIP complex. The morphology images indicate that the decomposition of the Cu(II)–AmIP complex in the composite ink produces metallic copper, which can fill the interstitial regions between the Cu flakes. As a result, the connected Cu flakes on cellulose paper can serve as a good continuous electrical pathway.

### 3.2. Substrate effect on the resistivity of Cu films

We examined the substrate effect by comparing the resistivity of the Cu film on cellulose paper with that of the Cu film on PI; both films were obtained by heating the composite ink at 100°C for 15 s in air. The resistivity on cellulose paper was approximately 10 times lower than that on PI (i.e. 7.0 × 10^−5^ Ω cm on paper versus 7.9 × 10^−4^ Ω cm on PI). Such a large effect of the substrate on the resistivity is attributed to the different uniformity of the Cu films after sintering. We observed the Cu films on both the paper and PI substrates by transmitted-light microscopy. If large holes are present in the Cu films, light from the opposite side will pass through the film, resulting in light spots in the image. The transmitted-light microscopy images show that numerous holes are present in the Cu film on PI (electronic supplementary material, figure S4a), whereas fewer holes are present in the Cu film on cellulose paper (electronic supplementary material, figure S4b). The gas released from the decomposition of the MOD ink can reduce the quality of the produced Cu film [31]. As a result, many holes (caused by the gas release) are observed in the Cu films on PI substrates. By contrast, such gas can be released from the Cu film through the porous paper substrate, leading to the production of a more continuous conductive Cu film on the paper substrate.

The composite ink used in this study contains ethylene glycol and glycerol as solvents. These alcohol-based solvents can wet cellulose paper. When the alcohol-based MOD ink in the composite ink partially penetrates the porous paper, large Cu flakes accumulate on the paper surface. The TG curves before and after the paper filtration indicate that the Cu content of the composite ink increased from 55 wt% to 75 wt% after filtrations of the composite ink (electronic supplementary material, figure S5). The high Cu content of the composite ink increases the packing density of Cu flakes in the sintered Cu film on the paper. The SEM reflection image of a cross section of the Cu films on cellulose paper confirmed the filtration-induced accumulation of Cu flakes on the paper (figure 3*c*,*d*). Owing to the high crystallinity of the Cu flakes, the reflection images clearly show their distribution in the sintered Cu films. Relative to the Cu film on PI, the Cu film on cellulose paper exhibits closer stacking of the Cu flakes, which serves to decrease the resistivity of the Cu film on the paper compared with that of the Cu film on PI.

We also examined the paper substrate effect by comparing different filtration papers with different filtration speeds (grades 3 and 4). The grade 4 is a fast-flow filter paper with a flow rate of 37 s/100 ml, whereas the grade 3 is a slow-flow filter paper with a flow rate of 325 s/100 ml. The use of fast-flow paper (grade 4) resulted in low electrical resistivity for Cu films deposited onto the paper substrate from the Cu-based composite ink and sintered at 100°C for 15 s in air (1 × 10^−4^ Ω cm for grade 3 and 7 × 10^−5^ Ω cm for grade 4). The fast flow rate of the MOD ink by the filter paper is desirable for the filtration-induced accumulation of Cu flakes on the paper surface in the Cu-based composite ink of the MOD ink and Cu flakes.

### 3.3. Bending stability of sintered Cu films on cellulose paper

**Figure 4:**
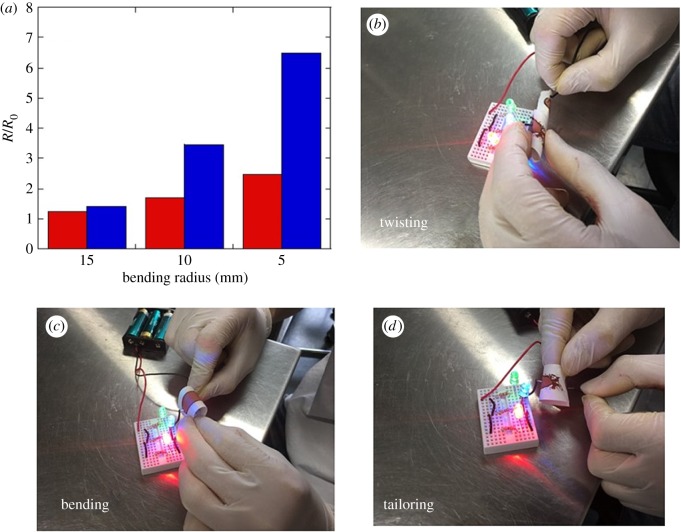
(*a*) The *R*/*R*_0_ values obtained from bending tests with bending radii of 5, 10 and 15 mm for the Cu films on cellulose paper (red) and PI (blue), as prepared from the composite ink and sintered at 100°C for 15 s in air. The photographs showing light-emitting diodes demonstrate the high bending durability of the Cu film on cellulose paper after the bending tests, with excellent mechanical capabilities of (*b*) twisting, (*c*) bending and (*d*) tailoring.

The bending stability of sintered Cu films on cellulose paper is an important factor for their practical application in flexible paper devices. The bending tests of the Cu films deposited onto cellulose paper and PI substrates and sintered at 100°C for 15 s in air were conducted with bending radii of 5, 10 and 15 mm. The *R*/*R*_0_ ratio was calculated after 1000 bending cycles, where *R*_0_ is the initial resistance and *R* is the resistance measured after the bending test. The resistance change (*R*/*R*_0_ ratio) of the Cu film on the paper was lower than that of the film on PI. After 1000 bending cycles with a bending radius of 5 mm, the *R*/*R*_0_ value of the Cu film on paper was 2.5, whereas that for the film on PI was 6.5 (figure 4*a*). As shown in figure 4*b–d*, the lighting of produced light-emitting diodes demonstrates high mechanical durability against bending, twisting and tailoring for Cu films on cellulose paper after the bending tests. The anchor effect of a porous paper surface to a Cu film and the filtration-induced accumulation of Cu flakes as films with fewer defects on cellulose paper are both likely to increase the bending stability. However, after 1000 bending cycles, the resistance of the Cu film on the paper was 2.5 times greater than its resistance before the test; thus, room exists for improvement. The increased resistance after the bending test can arise from crack initiation in the Cu films due to repeated tensile loading.

**Figure 5:**
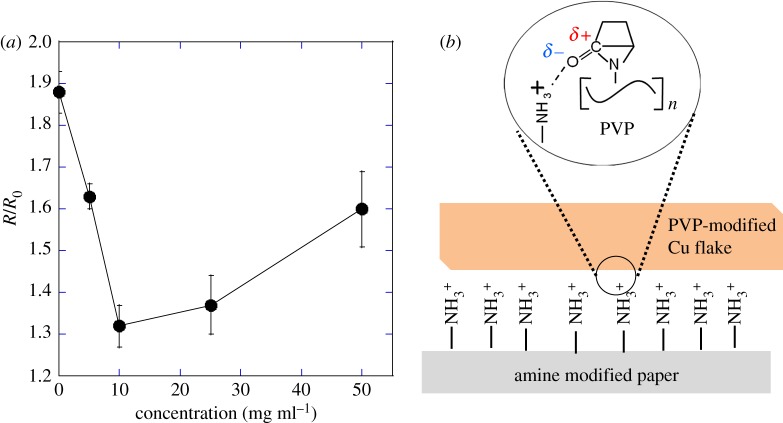
(*a*) The *R*/*R*_0_ values obtained from bending tests with a bending radius of 5 mm for the composite ink of PVP-modified Cu flakes deposited onto amine-modified cellulose paper and sintered at 100°C for 15 s in air. (*b*) Schematic showing the ion–dipole interaction between the ketone group of PVP-modified Cu flakes and the protonated amine group of a cellulose paper surface to give a Cu film with superior bending stability.

To further enhance the bending stability of the sintered Cu films on cellulose paper, we conducted the surface modification of cellulose paper and Cu flakes. Firstly, amino groups were introduced onto the cellulose paper using a silane coupling method. As a result, the *R*/*R*_0_ value decreased from 2.5 on the non-modified paper to 1.9 on the amine-modified paper after 1000 bending cycles with a bending radius of 5 mm, indicating that the amine modification of cellulose paper strengthened the bending stability. Next, Cu flakes were further modified with PVP and the extent of modification was controlled by varying the concentration of PVP used in the preparation from 5 mg ml^−1^ to 50 mg ml^−1^. The *R*/*R*_0_ values depend on the PVP concentration, and the minimum value was obtained at the concentration of 10 mg ml^−1^ (figure 5*a*). In the optimized condition, the resistance of the Cu film on the paper showed only a 1.3-fold and 1.1-fold increase at a bending radius of 5 mm and 15 mm, respectively, after 1000 bending cycles. This excellent bending stability is explained by the ion–dipole interaction between the ketone group of the PVP-modified Cu flakes and the protonated amine group of the paper surface [45]; the amine is protonated by the protons from formic acid or oxalic acid in the ink (figure 5*b*). The results show how the bending stability of Cu films on cellulose paper can be improved. Notably, the resistivity of the Cu film increased with increasing PVP concentration (e.g. 1.4 × 10^−4^ Ω cm at a concentration of 50 mg ml^−1^ PVP), whereas the increase in resistivity of the Cu film was small at the low concentration of 10 mg ml^−1^ PVP (9 × 10^−5^ Ω cm).

### 3.4. Suppression of Cu oxide formation in an air-sintering process by antioxidant additives and a low sintering temperature of 100°C

Finally, we describe the optimization of the Cu-based composite ink to achieve air-sinterability. The present Cu composite ink consists of a Cu(II)–AmIP complex and formic-acid-protected Cu flakes. The self-reducing action from formic-acid-capped Cu flakes and the AmIP were essential to suppress the copper oxidation during the air-sintering process. The formic acid not only decreases the sintering temperature of the Cu particles but also increases the conductivity of the sintered Cu particles. The XRD patterns demonstrate that the surface Cu oxide layer of Cu flakes was eliminated after the formic acid treatment (electronic supplementary material, figure S6). The chemical reduction of copper oxide by reducing agents during the sintering process can produce very active Cu particles that can merge with each other readily to reduce the surface free energy [16,25,46]. When no formic acid treatment was applied to the Cu flakes in the composite ink, the sintered Cu film obtained after sintering at 100°C for 15 s in air showed dominant oxidation (electronic supplementary material, figure S7) and the resistivity of the resultant Cu film was high (greater than 10^6^ Ω cm). These results indicate that the reducing action of the formic-acid-treated Cu flakes produced a conductive Cu film at low sintering temperatures in air.

Notably, prolonged heating times greater than 15 s increased the resistivity of the sintered Cu film, as shown in figure 2*b*. The rate of increase of the resistivity was higher at higher heating temperatures because of the enhanced copper oxidation. The low sintering temperature of 100°C is beneficial in terms of the slow growth rate of copper oxide in air. The rate of increase of the resistivity became small when the heating time was longer than 60 s, as shown in figure 2*b*. The initial growth rate of copper oxide islands has been reported to be high, whereas the coalescence of subsequent islands slows the oxidation rate (i.e. self-limiting oxidation of copper) [47]. In figure 2*b*, the initial rapid growth of the resistivity at heating times less than 60 s can be explained by the growth of copper oxide islands; the slow increase of the resistivity thereafter may originate from the self-limiting oxidation of copper in the coalesced islands.

To clarify the surface oxidation state resulting from the self-limiting oxidation of sintered Cu films in air, we used AES to examine the surface oxidation of Cu films sintered at 100°C for 60 s in air. The XPS chemical shift between Cu electron binding energies in CuO and Cu_2_O is very small, whereas the Cu Auger electron signals are well separated. The AES spectrum, as shown in electronic supplementary material, figure S8, indicates the surface oxidation state of the sintered Cu film. From the separation of the Cu-LMM peak, we estimated the ratio of surface oxidation species on the surface of Cu films as follows: Cu(0) = 26%, Cu(I) = 69% and Cu(II) = 5%. Thus, the self-limiting oxidation state of Cu films sintered in air at a low temperature of 100°C is mainly cupric oxide (Cu_2_O).

## 4. Conclusion

We proposed a novel, simple and effective Cu-based composite ink to produce conductive Cu films on cellulose paper under air-sintering conditions at 100°C for 15 s. To fabricate air-sinterable Cu-based inks at low temperatures, the following steps were developed for preparing the ink: (i) a composite ink comprising a MOD ink and Cu flakes was prepared; (ii) filtration-induced stacking of Cu flakes on cellulose paper and subsequent formation of a sintered Cu film with fewer defects were carried out; (iii) the combined reducing action of the formic-acid-treated Cu flakes and the MOD ink produced a conductive Cu film at low sintering temperatures in air. We also proposed a strategy to improve the bending stability of sintered Cu films on cellulose paper by exploiting the ion–dipole interaction between the ketone groups of PVP-modified Cu flakes and the amino groups of the paper surface. Under the optimized condition, the resistance of the Cu film on amine-modified cellulose paper was increased only 1.3-fold and 1.1-fold at bending radii of 5 mm and 15 mm, respectively, after 1000 bending cycles.

## Supplementary Material

Filtration-induced production of conductive/robust Cu films on cellulose paper by low-temperature sintering in air

## Data accessibility

Data supporting this study have been uploaded as the electronic supplementary material.
