# Supplementary material for: Filtration-induced production of conductive/robust Cu films on cellulose paper by low-temperature sintering in air
Source: R Soc Open Sci. 2018 Jul 4;5(7):172417. doi: 10.1098/rsos.172417 (PMC6083705; doi:10.1098/rsos.172417)
Supplement: Filtration-induced production of conductive/robust Cu films on cellulose paper by low-temperature sintering in air [file rsos172417supp1.pdf]

ROYAL SOCIETY
OPEN SCIENCE

R. Soc. open sci.
doi:10.1098/not yet assigned

Electric supplementary material

Filtration-induced Production of Conductive/Robust Cu films on Cellulose Filter Paper by Low-Temperature Sintering in Air

Shintaro Sakurai, Yusuke Akiyama, Hideya Kawasaki*

Faculty of Chemistry, Materials and Bioengineering, Kansai University, 3-3-35 Yamate-cho, Suita 564-8680, Japan

ROYAL SOCIETY
OPEN SCIENCE

R. Soc. open sci.
doi:10.1098/not yet assigned

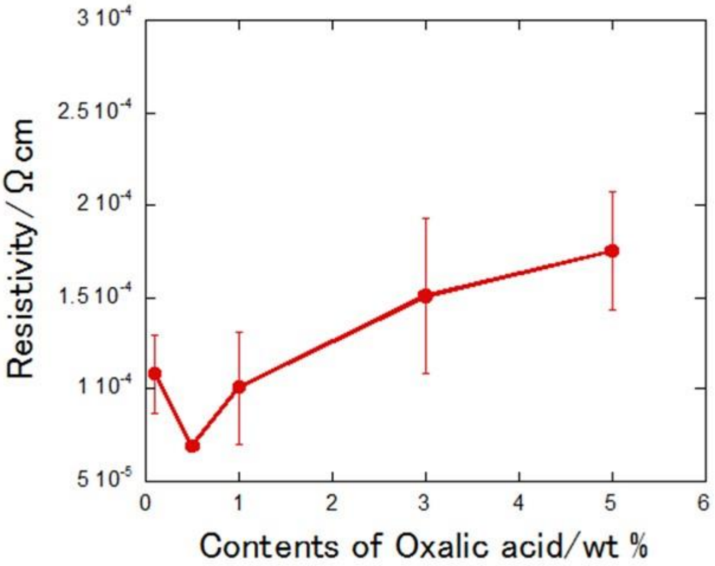

Figure S1 Electrical resistivity of Cu films on paper sintered at 100 °C for 15 s in air; the films were produced from a composite Cu ink with the addition of oxalic acid in various concentrations.

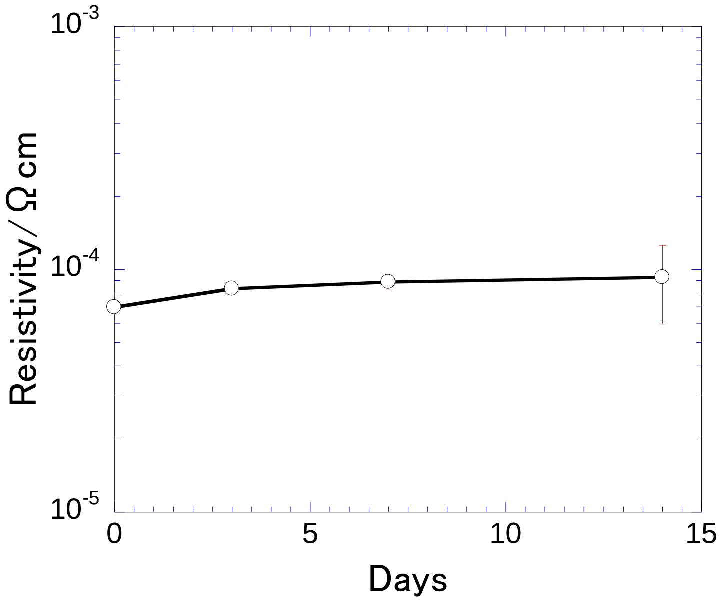

Figure S2 Electrical resistivity of Cu films produced by deposition of Cu ink onto paper and sintering at 100 °C for 15 s in air, plotted as a function of the number of days the ink was exposed to the air.

ROYAL SOCIETY
OPEN SCIENCE

R. Soc. open sci.
doi:10.1098/not yet assigned

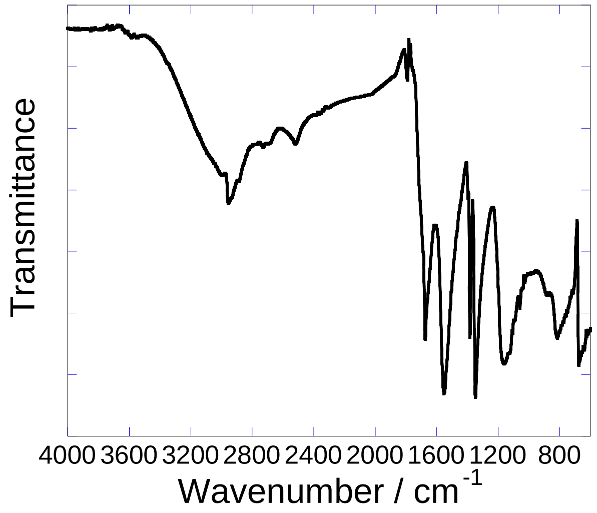

Figure S3 FT-IR spectrum of formic acid-protected Cu flakes: OH stretching band of COOH (3300–2500 cm^{-1}), C=O stretching band of COOH (1700 cm^{-1}) and O–H deformation vibration of COOH (1000–850 cm^{-1}).

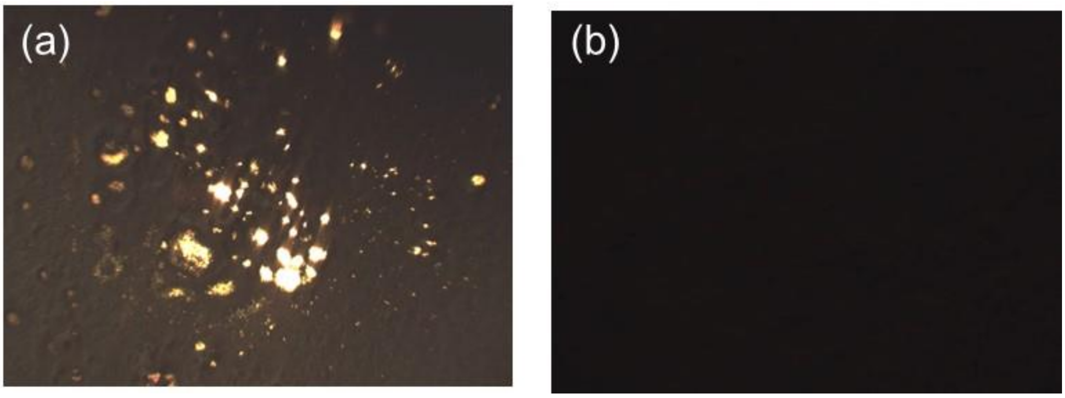

Figure S4 Transmitted-light microscopy images of Cu films on (a) polyimide film and (b) paper substrate; the films were sintered at 100 °C for 15 s in air.

ROYAL SOCIETY
OPEN SCIENCE

R. Soc. open sci.
doi:10.1098/not yet assigned

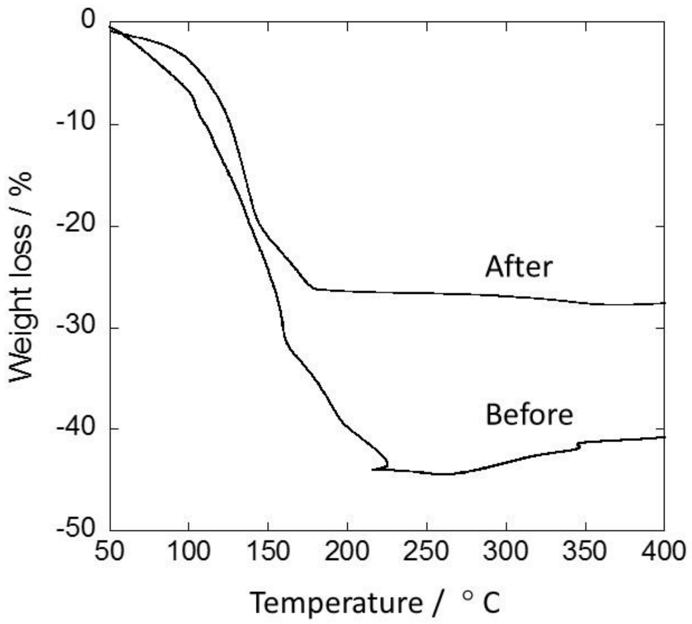

Figure S5 TG curves of composite Cu ink before and after the filtration through paper

.

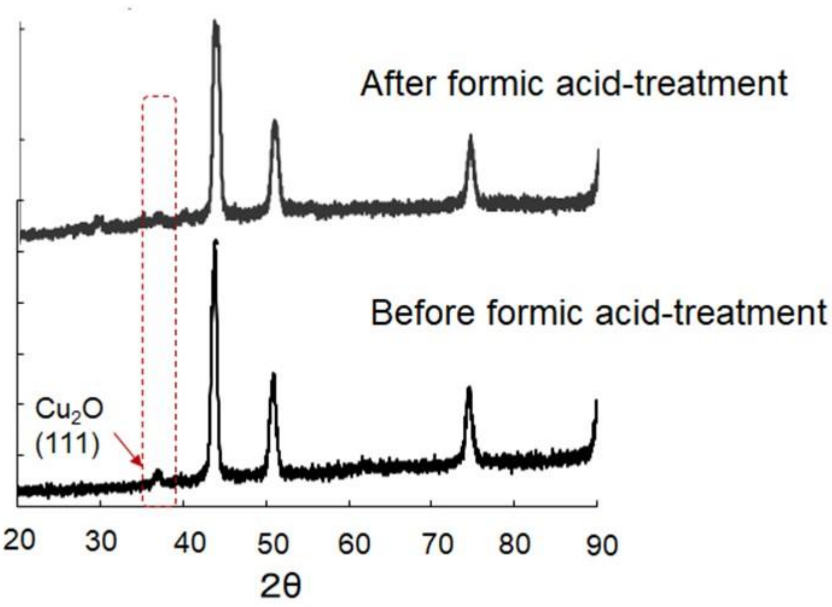

Figure S6 XRD pattern of Cu flakes before and after the formic acid treatment.

ROYAL SOCIETY
OPEN SCIENCE

R. Soc. open sci.
doi:10.1098/not yet assigned

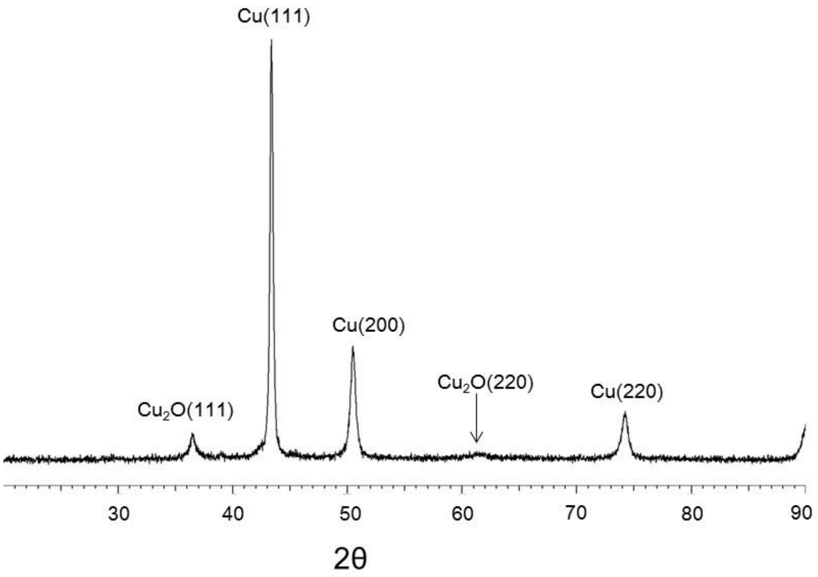

Figure S7 XRD pattern of a Cu film sintered at 100 °C for 15 s in air; the film was prepared from the composite ink with no formic acid treatment of Cu flakes and with no addition of oxalic acid.

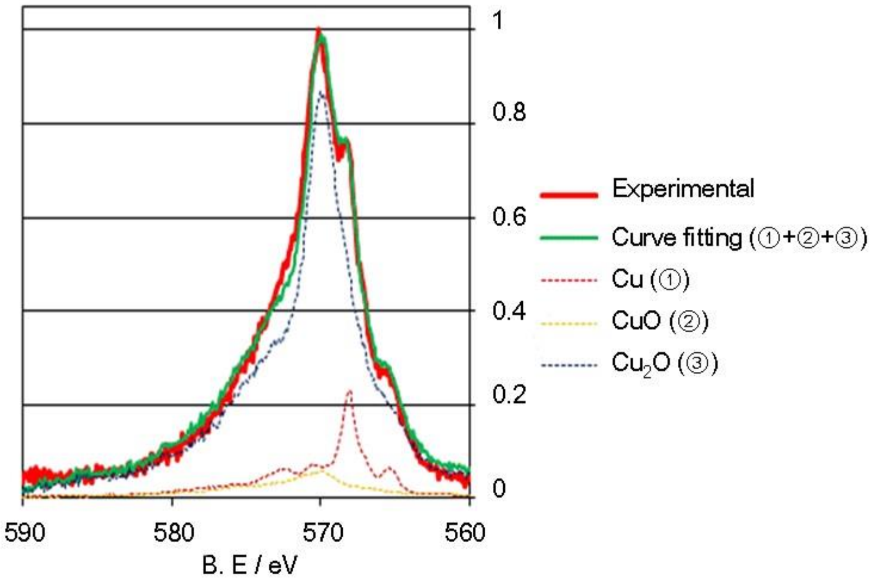

Figure S8 AES spectrum of the Cu film sintered at 100 °C for 60 s under an air atmosphere.
